# Artificial intelligence-based coronary computed tomography angiography quantification of atherosclerosis burden: comparison with intravascular ultrasound in the INVICTUS Registry

**DOI:** 10.1007/s00330-026-12412-y

**Published:** 2026-03-05

**Authors:** Rine Nakanishi, Ryo Okubo, Hitoshi Matsuo, Yoshihiro Sobue, Umihiko Kaneko, Hideyuki Sato, Shinichiro Fujimoto, Yui Nozaki, Takashi Kajiya, Toru Miyoshi, Keishi Ichikawa, Mitsunori Abe, Toshiro Kitagawa, Hiroki Ikenaga, Kazuhiro Osawa, Mike Saji, Nobuo Iguchi, Gaku Nakazawa, Kuniaki Takahashi, Takeshi Ijichi, Hiroshi Mikamo, Akira Kurata, Masao Moroi, Raisuke Iijima, Daniel Bandeira, Abigail Demuyakor, Helen Parise, Shant Malkasian, Gary S. Mintz, Alexandra J. Lansky, James P. Earls, Daniel Chamié

**Affiliations:** 1 Department of Cardiovascular Medicine, Toho University Graduate School of Medicine, Toho University Omori Medical Center, Tokyo, Japan; 2 Division of Cardiovascular Medicine, Department of Internal Medicine, Toho University Faculty of Medicine, Toho University Omori Medical Center, Tokyo, Japan; 3 Department of Cardiovascular Medicine, Gifu Heart Center, Gifu, Japan; 4 Sapporo Cardiovascular Clinic, Hokkaido, Japan; 5 Edogawa Hospital Tokyo, Tokyo, Japan; 6 Department of Cardiovascular Biology and Medicine, Juntendo University, Graduate School of Medicine, Tokyo, Japan; 7 Tenyoukai Central Hospital, Kagoshima, Japan; 8 Department of Cardiovascular Medicine, Dentistry and Pharmaceutical Sciences, Okayama University Graduate School of Medicine, Okayama, Japan; 9 Yotsuba Circulation Clinic, Matsuyama, Japan; 10 Department of Cardiovascular Medicine, Hiroshima University Graduate School of Biomedical and Health Sciences, Hiroshima, Japan; 11 Department of General Internal Medicine 3, Kawasaki Medical School General Medical Center, Okayama, Japan; 12 Okayama Red-Cross Hospital, Okayama, Japan; 13 Department of Cardiology, Sakakibara Heart Institute, Tokyo, Japan; 14 Department of Cardiology, Kindai University Faculty of Medicine, Osaka, Japan; 15 Department of Cardiology, Tokai University, School of Medicine, Isehara-shi, Japan; 16 Department of Cardiology, Toho University Sakura Medical Center, Chiba, Japan; 17 Department of Cardiology, NHO Shikoku Cancer Center, Matsuyama, Japan; 18 Department of Radiology, Ehime University Graduate School of Medicine, Matsuyama, Japan; 19 Department of Cardiovascular Medicine, Toho University Ohashi Medical Center, Tokyo, Japan; 20 Section of Cardiovascular Medicine, Department of Internal Medicine, Yale School of Medicine, New Haven, CT USA; 21 Yale Cardiovascular Research Group, Yale School of Medicine, New Haven, CT USA; 22 Department of Radiology, University of California, San Diego, La Jolla, CA USA; 23 Cardiovascular Research Foundation, New York, NY USA; 24 George Washington University School of Medicine and Health Sciences, Washington, DC USA

**Keywords:** Coronary artery disease, Atherosclerosis, Artificial intelligence, Computed tomography angiography, Ultrasonography

## Abstract

**Objectives:**

Automated artificial intelligence (AI)-based assessment of atherosclerosis burden applied to coronary computed tomography angiography (CCTA) can optimize image processing times, standardize interpretation, and minimize inter-observer variability. We investigated the diagnostic utility of AI-based CCTA quantification (AI-QCT) of coronary atherosclerosis in coronary segments co-registered with intravascular ultrasound (IVUS) of diseased and non-diseased segments.

**Materials and methods:**

Patients who underwent CCTA and IVUS in the INVICTUS registry (ClinicalTrials.gov: NCT04066062) were enrolled. Images were analyzed by independent core laboratories blinded to each modality’s findings. Vessel external elastic membrane (EEM), lumen, plaque volumes, plaque burden, and percent atheroma volume (PAV) were quantified in whole co-registered segments and subsegments containing non-calcified and low-attenuation plaques. A calcium index was calculated for the whole co-registered segment.

**Results:**

A total of 108 vessels from 85 patients were included. Pearson’s correlation demonstrated strong associations between AI-QCT and IVUS in quantifying the EEM volume (r = 0.899), lumen volume (r = 0.943), and plaque volume (r = 0.833), length-normalized PAV (r = 0.851), and calcium index (r = 0.960) in the whole-segment analysis. Strong correlations were seen for vessel, lumen, and plaque volumes in non-calcified (Pearson’s coefficient: 0.95, 0.97, and 0.83, respectively) and low-attenuation (Pearson’s coefficient: 0.90, 0.86, and 0.86, respectively) plaque segments. The minimum lumen area was 0.61 ± 1.18 mm^2^ (95% CI, −0.83 to −0.38) smaller by AI-QCT than IVUS, with a similar lumen area stenosis (mean difference, 1.26 ± 24.17; 95% CI, −3.37 to 5.90).

**Conclusions:**

AI-QCT quantification of atherosclerosis burden showed high correlations and close agreement with IVUS in whole-segment and segments with non-calcified and low-attenuation plaques.

**Key Points:**

***Question*** *Coronary atheroma burden is a powerful predictor of cardiovascular events. Can AI-based coronary CT angiography (CCTA) accurately quantify atherosclerotic burden across the full disease spectrum when compared with intravascular ultrasound (IVUS)?*

***Findings*** *AI-based CCTA quantification (AI-QCT) showed strong correlations with IVUS for plaque volume, burden, and calcium across whole coronary segments, including non-calcified and low-attenuation plaques.*

***Clinical relevance*** *AI-QCT provides rapid, automatic, and accurate atherosclerosis quantification without reader-dependent variability, enabling standardized cardiovascular risk assessment, treatment monitoring, and therapeutic decision-making across all disease severity spectrum in routine clinical practice.*

**Graphical Abstract:**

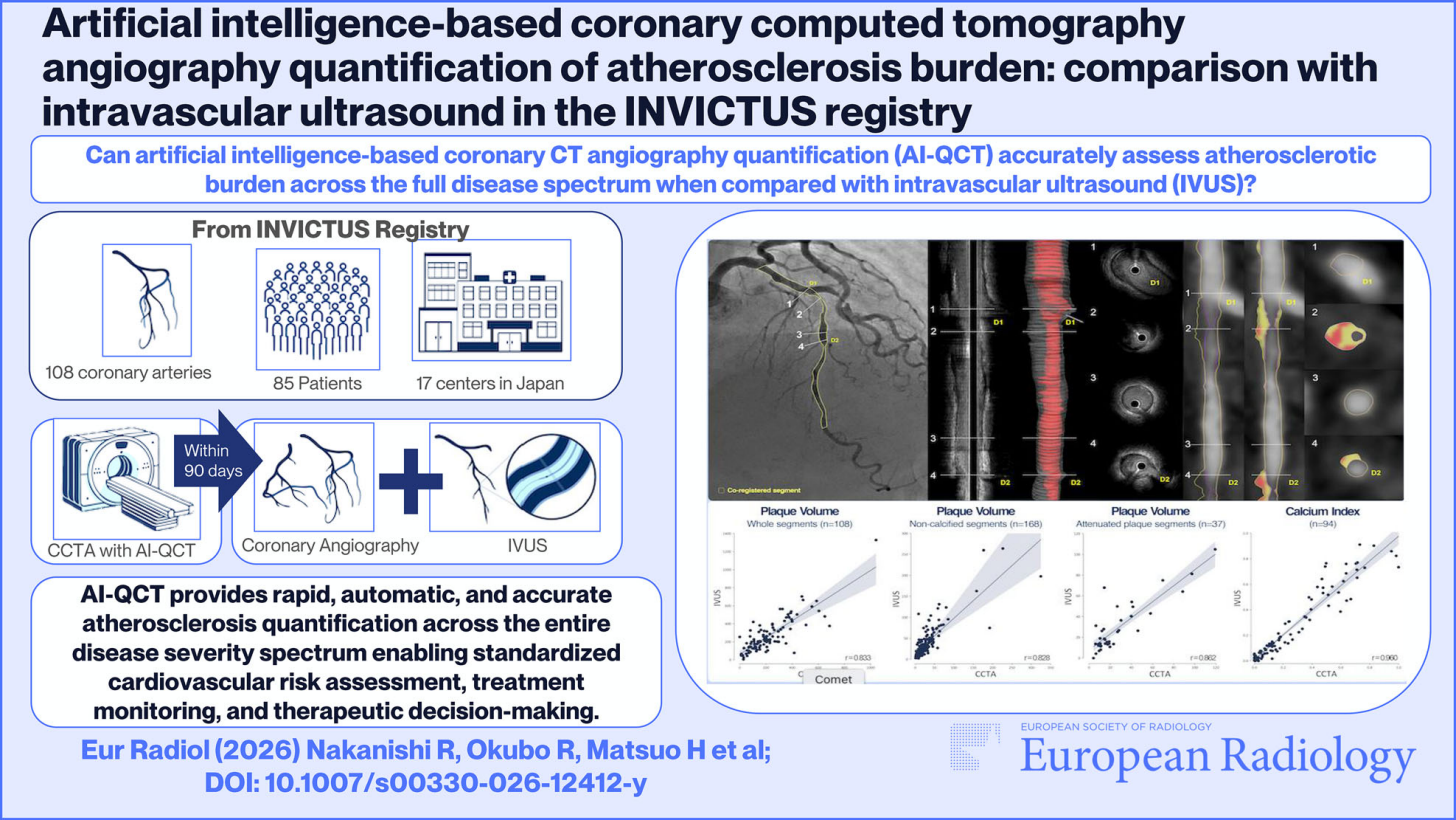

## Introduction

Recognition of the prognostic significance of coronary plaque burden beyond luminal obstruction [1–3] sparked advancements in quantifying total coronary atheroma burden. Initially estimated by calcium scoring surrogates, atheroma burden quantification has progressed from a qualitative assessment to a semi-quantitative standard [4, 5]. However, semi-automatic quantification is time-consuming, expertise-dependent, and carries significant inter-observer variability [6].

Artificial intelligence (AI) can expedite an accurate and reproducible quantification of atherosclerosis with minimal human interaction, potentially standardizing risk stratification independent of reader expertise, facilitating objective monitoring of disease progression or regression, and providing reproducible metrics for therapeutic decision-making. Few studies correlated AI-based plaque quantification on coronary computed tomography angiography (CCTA) images with intravascular ultrasound (IVUS) and have been limited to lesions with high plaque burden (typically > 40%) [7, 8]. While this lesion-centric approach maximizes noninvasive-invasive correlations, it fails to validate AI-based quantification of total coronary atheroma burden and overlooks the prognostic importance of mild atherosclerosis [9]. Quantitative plaque analysis must be accurate not only for high plaque burden lesions, but for the entire coronary tree, including normal, low, and high plaque burden segments.

In the current analysis of the Multicenter Registry of INVasive and non-Invasive imaging modalities to compare coronary Computed Tomography angiography, intravascular Ultrasound, and optical coherence tomography for the determination of severity, volume, and type of coronary atherosclerosiS (INVICTUS), we investigated the diagnostic utility of an AI-based CCTA (AI-QCT) quantification of coronary atherosclerosis across the whole spectrum of atheroma burden, including diseased and non-diseased continuous vascular segments.

## Materials and methods

### Study design and patient population

The INVICTUS registry design was previously published [10]. Briefly, INVICTUS is a multicenter registry conducted at 17 Japanese centers, collecting retrospective and prospective data from January 2011 to December 2021. Prospective patients provided written informed consent. For retrospective cases, opt-out forms were posted on each facility’s website and/or local sites to inform patients who did not wish their information included in the study. The study protocol conforms to the 1975 Declaration of Helsinki, was approved by the Toho University Omori Medical Center Ethics Committee (M24009_22063_21054_19012) and prospectively registered at ClinicalTrials.gov (NCT04066062).

The current study aimed to validate AI-QCT quantification of coronary atherosclerosis using IVUS as the invasive reference standard. A fraction of cases sharing CCTA and IVUS images were randomly selected from the INVICTUS registry database. We followed the STARD 2015 guidelines where applicable (Supplementary Table 1) [11]. Patients were eligible if they were 18 years of age or older, had coronary artery disease (CAD) identified at CCTA, were clinically stable, and subsequently underwent invasive coronary angiography or percutaneous coronary intervention with adjunctive use of IVUS no later than 3 months after CCTA. Patients were excluded if they could not give written informed consent in the prospective phase of the study, presented with acute ST-elevation myocardial infarction, were clinically unstable, were pregnant women, had an angiographically confirmed thrombus at the lesion site, did not have IVUS imaging before percutaneous coronary intervention, required balloon angioplasty or any lesion manipulation before IVUS imaging, IVUS images were acquired with manual pullbacks, if there was a previous stent in the target vessel segment, or if the time lapse between CCTA and IVUS imaging was longer than 3 months.

### CCTA image acquisition

CCTA was performed with 64-slice multidetector CT scanners (SOMATON Definition Flash and SOMATOM Force, Siemens; Aquilion ONE ViSION Edition and Aquilion Precision, Canon; Revolution, GE HealthCare; iCT, Philips Healthcare) per the SCCT guidelines [12]. Before CCTA, sublingual nitroglycerin tablets or spray (0.4–0.8 mg) were administered. Oral and/or intravenous beta-blockers were administered to reach a heart rate < 60 beats/min per local protocol, as needed. Scanning parameters were 70% to 80% of the R-R interval for prospective electrocardiogram-triggering studies and 35% to 80% for retrospective studies, collimation 64 × 0.625 mm, tube voltage 100–120 kV, and tube current 350–780 mA.

### IVUS image acquisition

IVUS images were acquired after administration of intracoronary nitroglycerin (200–400 mg) with commercially available 40-MHz (ViewIT, Terumo; TVC Insight, InfraRedx) or 60-MHz (OPTICROSS, Boston Scientific; AltaView, Terumo) mechanically rotating catheters. The imaging probe was advanced at least 10 mm distal to the target coronary lesion, then pulled back with an automated motorized device at 0.5 or 1.0 mm/s to the aorta-ostial junction or the maximum pullback length.

### Co-registration of CCTA and IVUS images

**Fig. 1:**
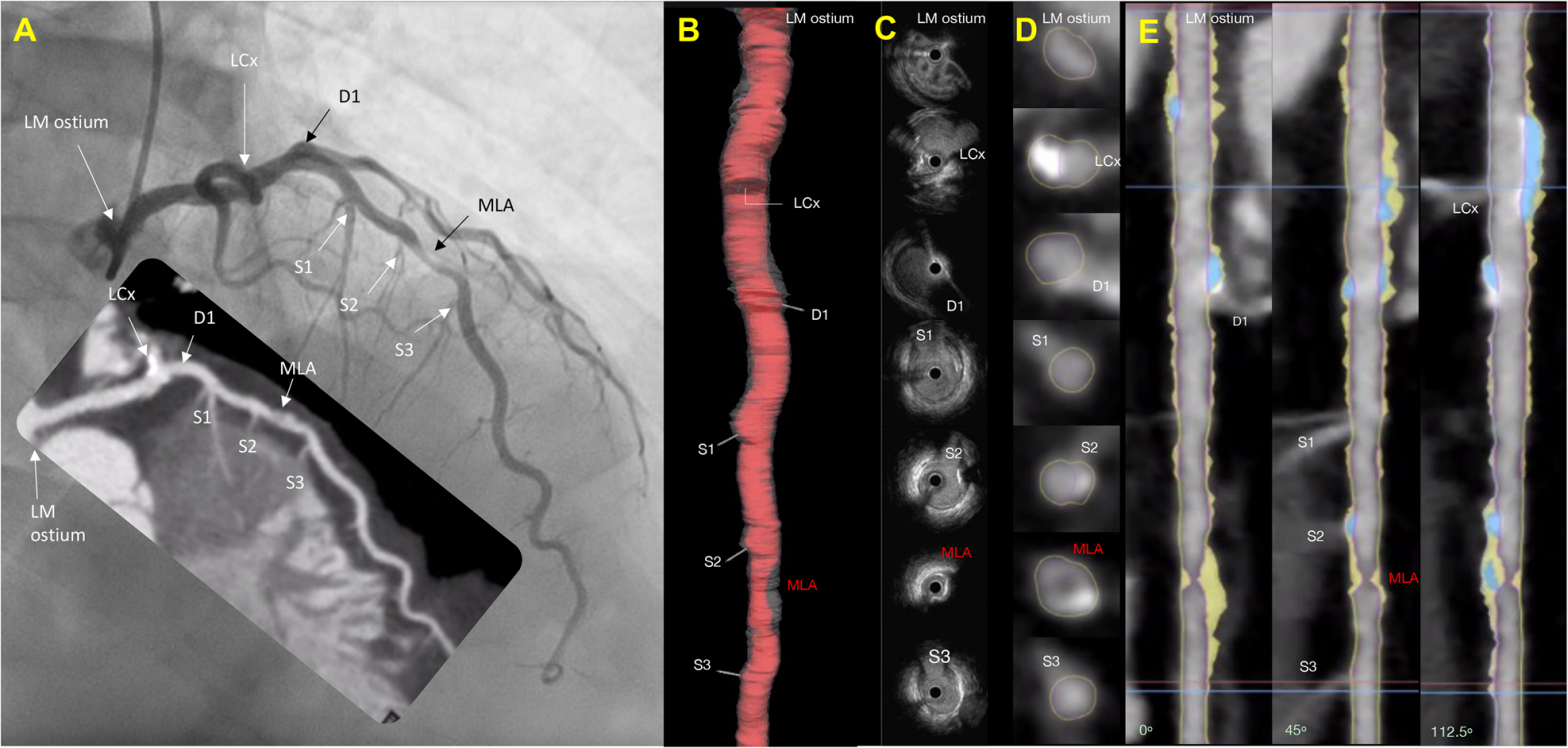
Example of multi-modality co-registration. First, the intravascular ultrasound (IVUS) core laboratory created a detailed map of anatomical landmarks (coronary ostium, side branches, calcifications, and stenoses) common to the angiography (**A**) and IVUS pullback (**B**, **C**). Next, the same landmarks were identified on coronary computed tomography angiography (CCTA) images (inset in **A** and **D**, **E**). The distance between the most proximal and distal co-registered landmarks defined the analysis segment, irrespective of the plaque burden. D (diagonal branches), LM (left main coronary artery), LCx (left circumflex artery), MLA (minimum lumen area), S (septal branches)

A detailed map of potential landmarks (eg, coronary ostium, side branches, calcifications, and stenoses) was performed on IVUS pullbacks and corresponding angiograms by an experienced analyst (D.C.) at the Yale IVUS Core Laboratory (Yale Cardiovascular Research Group). Next, 2 experienced analysts (D.C. and J.P.E.) conducted a tri-modality co-registration using the IVUS-angiography landmark mapping as a guide to match the same landmarks on CCTA images. Distances between landmarks were measured in the IVUS, angiography, and CCTA images for validation. The longest validated distance between landmarks common to CCTA and IVUS defined the whole analysis segment, which was not restricted to a lesion or stenosis (Fig. 1).

### Analysis of CCTA and IVUS images

CCTA and IVUS analyses of co-registered segments were performed at 0.5-mm intervals at Cleerly Labs (Cleerly Inc.) and Yale IVUS Core Laboratory, respectively. Core laboratories analyzed images independently and were blinded to each other’s findings.

CCTA images were analyzed with a commercially available and FDA-approved AI-QCT software (Cleerly Inc.), as previously described [13]. AI-QCT automatically generates vessel centerlines and contours of the inner lumen and outer vessel wall across all available phases, selecting the two highest-quality series for analysis. For each vessel, the optimal series is chosen based on minimal motion artifact and sufficient lumen opacification. The system automatically segments and labels each vessel, and plaques are identified and quantified based on Hounsfield unit (HU) values. To ensure accuracy, a qualified radiology technologist reviews the AI outputs for approval, as mandated by the FDA. Only CCTA images graded as excellent or good quality were included in the analysis. This workflow mirrors the clinically approved use of AI-QCT. The AI-QCT system has demonstrated excellent scan-rescan reproducibility in previous studies [14, 15].

The IVUS images were analyzed with the IvusPlus software version 1.1.0.0 (Pulse Medical), following the recommendations of the 2001 ACC Clinical Expert Consensus Document on Standards for Acquisition, Measurement, and Reporting of Intravascular Ultrasound Studies [16]. The external elastic membrane (EEM) and lumen contours were automatically determined and manually adjusted when necessary.

On CCTA and IVUS images, the EEM (outer vessel wall), lumen, plaque (EEM area − lumen area) areas, and plaque burden (plaque area / EEM area × 100) were calculated in each cross-section along the co-registered segment. The EEM, lumen, and plaque volumes, percent atheroma volume (PAV) [∑(EEM area − lumen area) / ∑ EEM area × 100], and length-normalized PAV were computed for the whole co-registered segments. A single, senior IVUS operator (D.C.) performed all IVUS analyses with excellent reproducibility (Supplementary Table 2).

On CCTA, a tissue with an area of ≥ 1 mm^2^ between the lumen and vessel wall defined a coronary plaque, which was classified into low-attenuation non-calcified (< 30 HU), non-calcified (30–350 HU), and calcified (> 350 HU) [10]. On IVUS, plaques were classified as highly attenuated, soft or echolucent, fibrotic, and calcified. Examples and definitions of plaque characterization by CCTA and IVUS are presented in Supplementary Table 3 and Supplementary Fig. 1. To allow a consistent comparison with CCTA, plaques characterized by IVUS as fibrotic, soft, and attenuated were deemed non-calcified. Of these, attenuated and soft plaques by IVUS and low-attenuation plaques by CCTA were further categorized as fibrofatty/lipid. Notably, IVUS can only detect the leading edge of calcium and attenuated plaques but does not allow for quantification of the areas and volumes of the calcified and attenuated components as CCTA does. Assuming the tissue behind superficial calcium or attenuation is entirely calcified or attenuated represents a fundamental error (Supplementary Figs. 2, 3). Therefore, we performed a subsegmental analysis reporting the entire plaque volume on vascular segments with non-calcified and attenuated plaques. We determined the arc of calcium using an electronic protractor centered in the lumen and the total length of calcification along the co-registered segments and calculated a validated calcium index [(total calcium length/co-registered vessel length) × (maximum calcium arc/360°)] [17] (Supplementary Fig. 4). For consistency, we computed the same calcium index on the CCTA images utilizing 500 HU as a threshold for calcium detection.

### Statistical analysis

A sample size of 107 vessels provided 90% power to detect ≥ 5% difference in PAV quantification between AI-QCT and IVUS, assuming a standard deviation of 15% and 0.05 significance level.

Categorical variables are presented as frequency and percentage, and continuous variables as mean ± SD with the 95% CI or median and IQR, when appropriate. Pearson’s correlation and linear regression analysis were conducted to determine the association between CCTA and IVUS metrics. Bland–Altman analysis was performed to assess the agreement between CCTA and IVUS.

The analyses were conducted on the whole co-registered segment and at a subsegmental level. All statistical analyses were conducted using SAS software version 9.4 (SAS Institute).

### Role of the sponsor

Cleerly Inc. sponsored the study by providing CCTA analysis through their academic core laboratory using the FDA-approved AI-QCT platform, ensuring that our validation reflects the actual clinical workflow.

To minimize the sponsor’s influence and bias, rigorous safeguards were implemented. The CCTA analysis at Cleerly’s core laboratory and IVUS analysis at Yale’s core laboratory were conducted independently, with each core lab blinded to the other. All data management, statistical analysis, and manuscript preparation were conducted independently at Yale University, with the senior and first authors maintaining full control over the study design, data interpretation, and manuscript content. Per standard academic-industry collaboration, the sponsor had access to the final manuscript before submission, but exercised no influence on the content, data interpretation, or the conclusions presented.

## Results

**Fig. 2:**
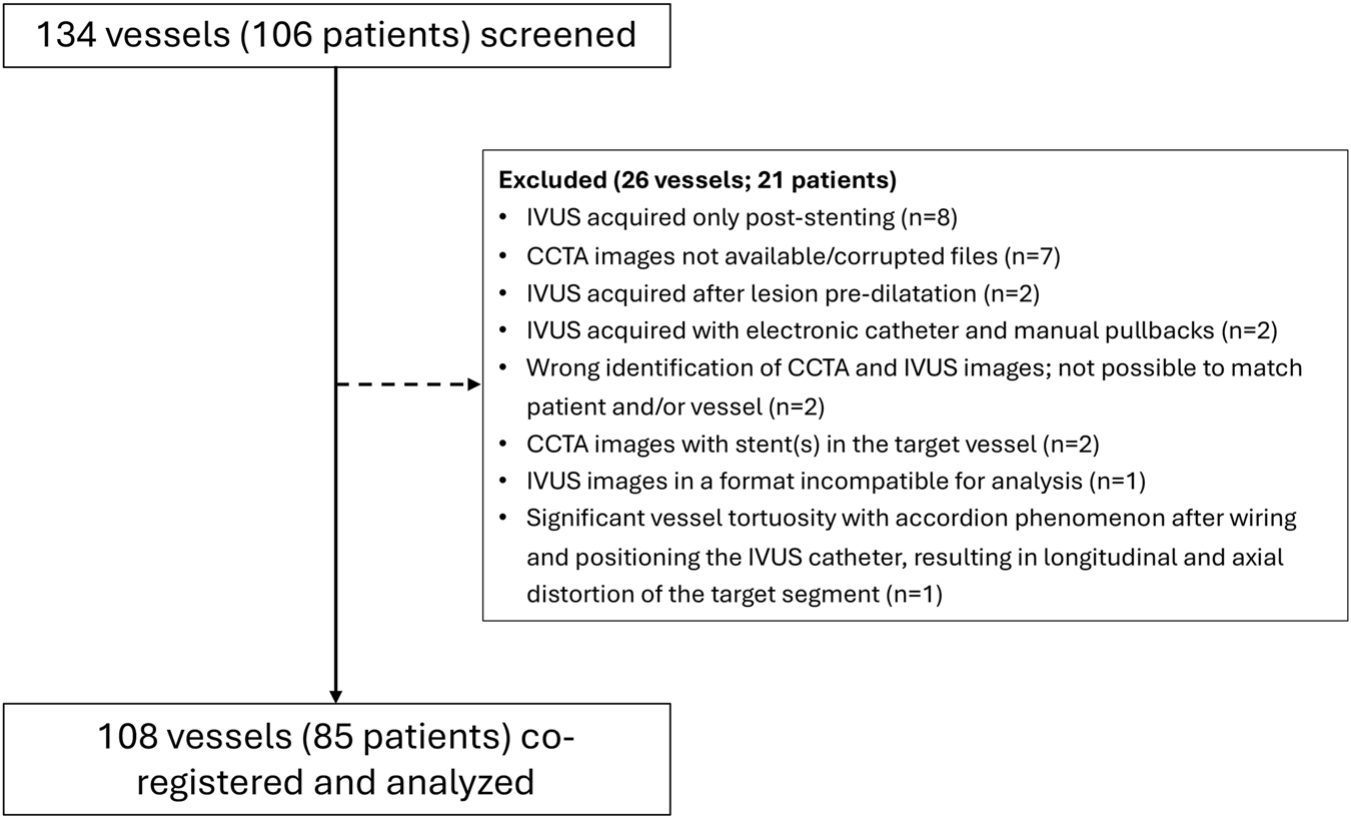
Study flowchart. Out of the 134 vessels screened, 108 passed the multi-modality co-registration and were included in the final analysis. CCTA, coronary computed tomography angiography; IVUS, intravascular ultrasound

After screening 134 vessels from 106 patients with matching angiography, IVUS, and CCTA images from the INVICTUS registry, we excluded 26 vessels (21 patients) due to image quality or co-registration issues. Final analysis included 108 co-registered coronary segments from 85 patients (Fig. 2), encompassing 235 subsegments (non-calcified, low-attenuation, calcified, or normal), providing comprehensive coverage of the atherosclerotic disease spectrum.

The mean interval between CCTA and IVUS acquisitions was 27.71 ± 21.03 days. Baseline demographics and clinical characteristics are in Table 1. The mean age was 71.1 ± 9.8 years, 71.8% were male, and one-third had diabetes. The left anterior descending coronary artery was the predominant target vessel (44.4%).

**Table 1:** Baseline demographic and clinical characteristics

	*N* = 85 patients
Age, years	71.1 ± 9.8
Male sex	61 (71.8%)
Height, cm	160.8 ± 9.2
Weight, kg	62.4 ± 13.0
Hypertension	57 (67.0%)
Hyperlipidemia	65 (76.5%)
Diabetes	33 (38.8%)
Family history of coronary artery disease*	6 (7.1%)
Ex-tobacco user (> 1 year)	24 (28.2%)
Current tobacco user (within 1 year)	21 (24.7%)
Prior cerebrovascular/transient ischemic attack	10 (11.8%)
Peripheral arterial disease	6 (7.1%)
History of heart failure	2 (2.4%)
Prior PCI	16 (18.8%)
Prior CABG	1 (1.2%)
Prior MI	9 (10.6%)
Target vessel	
Left main	5 (4.6%)
Left anterior descending artery	48 (44.4%)
Left circumflex artery	38 (35.2%)
Right coronary artery	17 (15.8%)

Values are mean ± SD or *n* (%)

*CABG* coronary artery bypass graft, *MI* myocardial infarction, *PCI* percutaneous coronary intervention

* Defined as coronary artery disease detected in a first-degree relative at age < 55 years for men and < 65 years for women

### Whole-segment AI-QCT and IVUS analysis

Table 2 presents AI-QCT and IVUS measures of vessel and atherosclerosis for the whole co-registered segments. The mean co-registered length was 38.3 ± 18.2 mm by AI-QCT and 38.5 ± 18.7 mm by IVUS. On a cross-section level, AI-QCT underestimated the vessel (EEM), lumen, and plaque areas by 2.9 ± 2.2 mm^2^, 1.63 ± 1.10 mm^2^, and 1.29 ± 1.98 mm^2^, respectively. There were no significant differences in the quantification of plaque burden (AI-QCT: 49.7 ± 14.3% vs IVUS: 51.3 ± 10.2; mean difference: −1.60 ± 12.12%, 95% CI: −3.91 to 0.71) or PAV (AI-QCT: 51.2 ± 14.0% vs IVUS: 51.3 ± 10.4%; mean difference: −0.09 ± 8.90%, 95% CI: −1.78 to 1.61). Bland–Altman analysis demonstrated close agreement between AI-QCT and IVUS. For PAV, the mean difference was −0.09% with 95% limits of agreement of −17.5% to +17.3%, indicating that in individual cases, AI-QCT measurements may differ from IVUS by up to approximately 17 percentage points. However, 94.4% of measurements fell within these limits, suggesting acceptable agreement for clinical application.

**Table 2:** AI-QCT and IVUS analysis of the whole co-registered segments

	CCTA		IVUS		Paired difference	
	Mean ± SD	95% CI	Mean ± SD	95% CI	Mean ± SD	95% CI
Total length, mm	38.3 ± 18.2	34.8 to 41.7	38.5 ± 18.7	35.0 to 42.1	−0.27 ± 2.34	−0.72 to 0.17
Mean EEM area, mm^2^	11.2 ± 4.4	10.3 to 12.0	14.0 ± 4.5	13.2 to 14.9	−2.9 ± 2.2	−3.3 to −2.4
EEM volume, mm^3^	428 ± 290	372 to 481	530 ± 350	464 to 597	−102.9 ± 155.4	−132.6 to −73.3
Mean lumen area, mm^2^	5.1 ± 2.0	4.8 to 5.5	6.8 ± 2.5	6.3 to 7.2	−1.63 ± 1.10	−1.84 to −1.42
Lumen volume, mm^3^	201 ± 144	173 to 228	256 ± 184	221 to 291	−55.5 ± 67.9	−68.4 to −42.5
Mean plaque area, mm^2^	6.0 ± 3.2	5.3 to 6.6	7.2 ± 2.9	6.7 to 7.8	−1.29 ± 1.98	−1.66 to −0.91
Plaque volume, mm^3^	227 ± 168	195 to 259	274 ± 186	239 to 310	−47.7 ± 103.5	−67.5 to −28.0
Mean plaque burden, %	49.7 ± 14.3	47.0 to 52.5	51.3 ± 10.2	49.4 to 53.3	−1.60 ± 12.12	−3.91 to 0.71
PAV, %	51.2 ± 14.0	48.5 to 53.8	51.3 ± 10.4	49.3 to 53.2	−0.09 ± 8.90	−1.78 to 1.61
Length-normalized PAV	1.83 ± 1.69	1.50 to 2.15	1.81 ± 1.49	1.52 to 2.09	0.02 ± 0.89	−0.15 to 0.19

*AI-QCT* artificial intelligence-based coronary computed tomography angiography quantification, *EEM* external elastic membrane, *IVUS* intravascular ultrasound, *PAV* percent atheroma volume

**Fig. 3:**
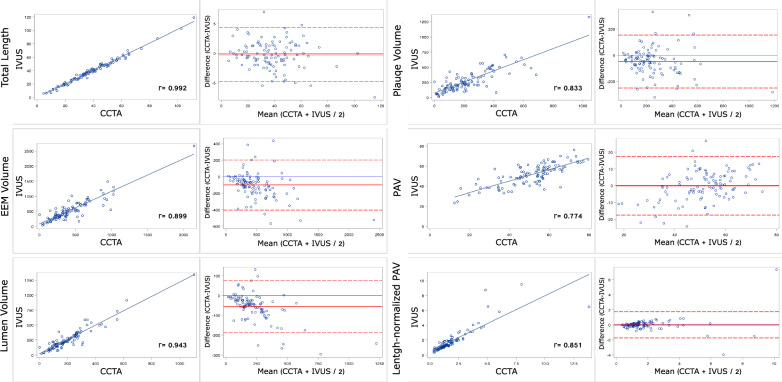
Scatterplots and Bland–Altman graphs for quantitative parameters in the whole-segment analysis. Scatterplots are presented with regression lines and Pearson’s correlation coefficient. In the Bland–Altman graphs, the solid red line indicates the mean difference (bias), and the dashed red lines indicate the 95% limits of agreement (1.96 × SD). CCTA, coronary computed tomography angiography; EEM, external elastic membrane; IVUS, intravascular ultrasound; PAV, percent atheroma volume

A Pearson’s correlation demonstrated strong associations between AI-QCT and IVUS for segment length (r = 0.992), EEM volume (r = 0.899), lumen volume (r = 0.943), plaque volume (r = 0.833), PAV (r = 0.774), and length-normalized PAV (r = 0.851) (Fig. 3).

**Fig. 4:**
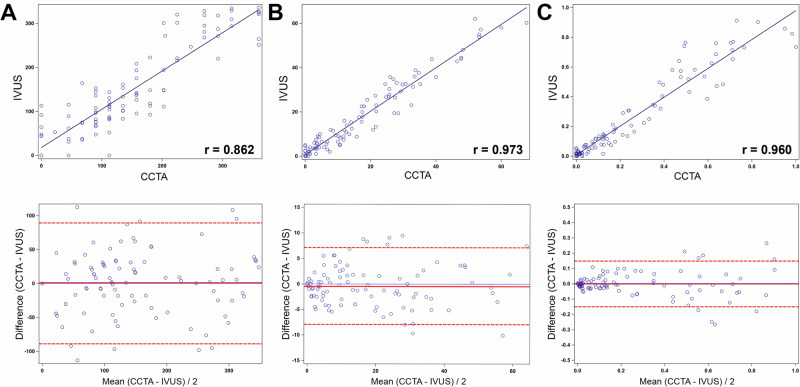
Scatterplots and Bland–Altman graphs for coronary calcium quantification. Scatterplots with regression lines, Pearson’s correlation coefficients, and Bland–Altman analysis of calcium angle (**A**), total calcium length (**B**), and calcium index (**C**). In the Bland–Altman graphs, the solid red line indicates the mean difference (bias), and the dashed red lines the 95% limits of agreement (1.96 × SD). CCTA, coronary computed tomography angiography; IVUS, intravascular ultrasound

AI-QCT and IVUS also demonstrated strong correlations for quantification of maximum calcium angle (r = 0.906), total calcium length (r = 0.973), and calcium index (r = 0.960) (Fig. 4).

### Analysis at the minimum lumen area (MLA) site

MLA sites on CCTA and IVUS were independently determined and not actively co-registered; the MLA sites were automatically determined during post-processing of the exported CCTA and IVUS data as the cross-section with the smallest lumen area within the whole co-registered segments in both imaging modalities. AI-QCT and IVUS located the MLA cross-section 25.5 ± 18.5 mm and 23.7 ± 17.9 mm distally to the most proximal landmark of the co-registered segment (mean difference, 1.79 ± 26.83 mm; 95% CI, −3.33 to 6.91 mm). MLA was 0.61 ± 1.18 mm^2^ (95% CI, −0.83 to −0.38) smaller by AI-QCT than IVUS. No significant differences existed for the EEM area (mean difference, −0.48 ± 3.87 mm^2^; 95% CI, −1.22 to 0.26), plaque area (mean difference, 0.15 ± 3.84 mm^2^; 95% CI, −0.58 to 0.88), plaque burden (mean difference, 3.6 ± 13.1%; 95% CI, 1.1 to 6.0), or lumen area stenosis (mean difference, 1.26 ± 24.17%; 95% CI, −3.37 to 5.90) (Table 3).

**Table 3:** AI-QCT and IVUS analysis at the minimum lumen area site

	CCTA		IVUS		Paired difference	
	Mean ± SD	95% CI	Mean ± SD	95% CI	Mean ± SD	95% CI
MLA, mm^2^	2.3 ± 1.9	2.0 to 2.7	3.0 ± 1.6	2.6 to 3.3	−0.61 ± 1.18	−0.83 to −0.38
MLA offset^a^, mm^2^	25.5 ± 18.5	22.0 to 29.0	23.7 ± 17.9	20.3 to 27.1	1.79 ± 26.83	−3.33 to 6.91
Mean EEM area, mm^2^	11.7 ± 5.6	10.6 to 12.8	12.2 ± 4.8	11.3 to 13.1	−0.48 ± 3.87	−1.22 to 0.26
Plaque area, mm^2^	9.4 ± 5.5	8.3 to 10.4	9.2 ± 4.2	8.4 to 10.0	0.15 ± 3.84	−0.58 to 0.88
Plaque burden, %	77.6 ± 17.8	74.2 to 81.0	74.1 ± 11.8	71.8 ± 76.3	3.6 ± 13.1	1.1 to 6.0
Lumen area stenosis, %	66.7 ± 27.6	61.4 to 72.0	65.3 ± 12.7	62.9 ± 67.7	1.26 ± 24.17	−3.37 to 5.90
Predominant plaque type^b^	*N* = 108		*N* = 108			
Calcified	41 (38.0%)		41 (38.0%)			
Non-calcified	66 (61.1%)		66 (61.1%)			
Fibrofatty/lipid	20 (30.3%)		21 (31.8%)			
Plaque rupture	0 (0%)		4 (19.0%)			
Normal vessel	1 (1.5%)		1 (1.5%)			

*AI-QCT* artificial intelligence-based coronary computed tomography angiography quantification, *EEM* external elastic membrane, *IVUS* intravascular ultrasound, *MLA* minimum lumen area

^a^ Distance from MLA site to the most proximal co-registered landmark

^b^ *n* (%)

CCTA and IVUS agreed in 99.1% of cases in classifying the predominant plaque type at the MLA (kappa, 0.98; 95% CI, 0.96–1.00); non-calcified plaque was most common (66/108; 61.1%) in both AI-QCT and IVUS MLA cross-sections. IVUS identified 4 plaque ruptures in 2 left anterior descending arteries and 2 right coronary arteries, not initially recognized by CCTA. Of these, 2 lesions were soft plaques by IVUS and had low attenuation by CCTA. The other 2 were ruptured superficial fibrotic and soft plaques over deep calcium by IVUS, not visible by CCTA due to intense calcium blooming.

### Subsegmental analysis

**Fig. 5:**
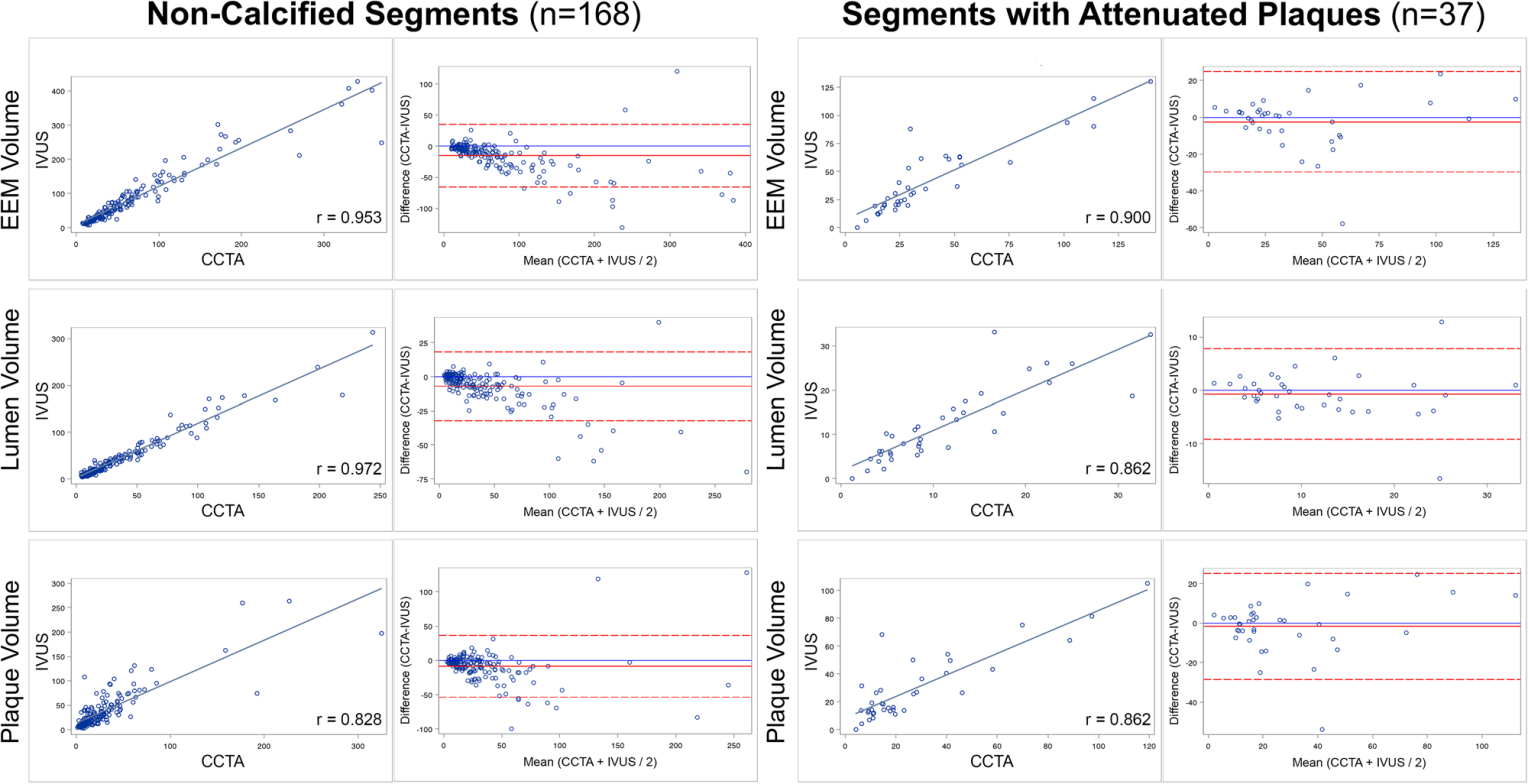
Scatterplots and Bland–Altman graphs for quantitative parameters in non-calcified and low-attenuation segments. Scatterplots are presented with regression lines and Pearson’s correlation coefficient. In the Bland–Altman graphs, the solid red line indicates the mean difference (bias), and the dashed red lines indicate the 95% limits of agreement (1.96 × SD). CCTA, coronary computed tomography angiography; EEM, external elastic membrane; IVUS, intravascular ultrasound

Table 4 and Fig. 5 present AI-QCT and IVUS analyses of segments with non-calcified and low-attenuation plaques and normal vessels.

**Table 4:** AI-QCT and IVUS subsegmental analysis

	CCTA		IVUS		Paired difference	
	Mean ± SD	95% CI	Mean ± SD	95% CI	Mean ± SD	95% CI
Non-calcified segments (*n* = 168 segments)						
EEM volume, mm^3^	64.6 ± 68.1	54.2 to 74.9	79.8 ± 80.4	67.6 to 92.1	−15.3 ± 25.8	−19.2 to −11.4
Lumen volume, mm^3^	36.3 ± 39.2	30.3 to 42.2	43.2 ± 47.0	36.0 to 50.3	−6.9 ± 12.8	−8.9 to −5.0
Mean plaque burden, %	42.4 ± 16.5	39.9 to 44.9	47.3 ± 11.7	45.5 to 49.1	−4.9 ± 14.4	−7.1 to −2.7
Plaque volume, mm^3^	28.3 ± 38.6	22.4 to 34.2	36.7 ± 39.5	30.6 to 42.7	−8.4 ± 23.0	−11.9 to −4.9
Attenuated plaque segments (*n* = 37 segments)						
EEM volume, mm^3^	38.8 ± 31.5	28.3 to 49.4	41.3 ± 30.9	31.0 to 51.6	−2.4 ± 14.0	−7.1 to 2.2
Lumen volume, mm^3^	11.3 ± 8.0	8.6 to 13.9	12.0 ± 8.5	9.1 to 14.8	−0.70 ± 4.35	−2.15 to 0.75
Mean plaque burden, %	66.1 ± 16.8	60.6 to 71.7	68.8 ± 10.3	65.4 to 72.3	−2.7 ± 12.9	−7.0 to 1.6
Plaque volume, mm^3^	27.6 ± 27.1	18.6 to 36.6	29.3 ± 24.3	21.2 to 37.4	−1.75 ± 13.76	−6.34 to 2.84
Normal vessel segments (*n* = 30 segments)						
EEM volume, mm^3^	52.9 ± 77.8	23.9 to 82.0	83.9 ± 118.0	39.8 to 127.9	−31.0 ± 60.4	−53.5 to −8.4
Lumen volume, mm^3^	44.7 ± 55.8	23.9 to 65.5	62.4 ± 87.4	29.7 to 95.0	−17.7 ± 42.4	−33.5 to −1.9
Intima-media burden, %*	4.8 ± 10.6	0.8 to 8.7	26.9 ± 4.3	25.4 to 28.5	−22.2 ± 10.6	−26.1 to −18.3
Intima-media volume, mm^3^	8.2 ± 30.1	−3.0 to 19.5	21.5 ± 31.0	9.9 to 33.0	−13.2 ± 25.3	−22.7 to −3.8

*AI-QCT* artificial intelligence-based coronary computed tomography angiography quantification, *EEM* external elastic membrane, *IVUS* intravascular ultrasound, *MLA* minimum lumen area

* Indicates how much of the EEM area is occupied by the intima-media complex, and is calculated as: (EEM area − lumen area) / EEM area × 100 in normal segments

There were 168 segments with non-calcified plaques. Pearson’s correlation analysis demonstrated a strong correlation between AI-QCT and IVUS for EEM (r = 0.953), lumen (r = 0.972), and plaque (r = 0.828) volumes.

Thirty-seven segments contained low-attenuation plaques. Strong correlations were found between AI-QCT and IVUS for the quantification of EEM (r = 0.900), lumen (r = 0.862), and plaque (r = 0.862) volumes.

Thirty segments involved normal vessels. CCTA lacks the resolution to resolve the coronary wall thickness in normal segments, superimposing the lumen and outer wall contours. Contrarily, IVUS detects the intima-media complex that separates the lumen from the EEM, and the analysis software misreports the EEM minus lumen difference as plaque (Supplementary Fig. 5). Due to these methodological differences, the IVUS quantified intima-media thickness obstructed the vessel area by 26.9 ± 4.3% in comparison with only 4.8 ± 10.6% by AI-QCT.

Strong correlations were also seen for plaque volumes in segments stratified by the CCTA-derived diameter stenosis and plaque stages (Supplementary Tables 4, 5, and Supplementary Figs. 6, 7).

## Discussion

The current analysis of the INVICTUS registry demonstrated that AI-QCT quantification of coronary atherosclerosis provided strong correlations and agreement with independent IVUS core laboratory quantifications across the same vascular segments, including total plaque volume, plaque volumes in non-calcified and attenuated plaque segments, and calcium index. Notably, AI-QCT accurately quantified lumen stenosis and identified the MLA location without manual input.

### Quantification of the total atherosclerotic burden

While prior multicenter studies have shown good agreement between deep-learning algorithms and expert CCTA readers or independent IVUS analysis, these focused mostly on lesions with high (> 40%) plaque burden [7, 8]. While these data are reassuring from a technical standpoint, the performance of AI-based CCTA quantification of coronary artery segments with low or no plaque burden remains unvalidated. The DECODE study [18] illustrated potential pitfalls when analysis is restricted to vascular regions with high plaque burden: 63% of studies were reclassified to a higher CAD stage, and 96% of patients were classified as having moderate-to-severe disease requiring significant treatment intensification, raising concerns about overtreatment that can add cost and potential harm to patients.

We expanded upon previous research by evaluating AI-based CCTA quantification of atheroma burden along continuous coronary segments, without restricting the analysis to segments with a certain plaque burden threshold, providing a broader and clinically meaningful assessment. Lumen and EEM (or outer vessel on CCTA) are key anatomical structures for quantifying coronary plaque area and volume. We found that AI-QCT slightly underestimated the EEM and lumen areas and volumes, which can be partly attributed to the lower resolution of CCTA in comparison with IVUS and the utilization of more potent vasodilation for IVUS (intracoronary nitroglycerin immediately before image acquisition) vs CCTA (sublingual nitroglycerin several minutes before image acquisition). However, this small, yet systematic underestimation did not affect atheroma burden quantification. Our data demonstrated a high concordance between AI-QCT and IVUS for plaque burden (mean difference: −1.60% ± 12.12; 95% CI: −3.91 to 0.71) and PAV (mean difference: −0.09% ± 8.90%; 95% CI: −1.78 to 1.61) quantification. When PAV was normalized by length, the difference between AI-QCT and IVUS was negligible (0.02 ± 0.89; 95% CI: −0.15 to 0.19).

### Atheroma burden according to plaque type segments

Plaque characterization by CCTA has prognostic implications. Low-attenuation non-calcified plaque volume by CCTA provides the strongest association for future myocardial infarction and MACE, independent of clinical risk factors, calcium score, or segment stenosis score [19, 20]. Previously, Omori et al [21] demonstrated high accuracy of AI-QCT for detecting low-attenuation plaque using near-infrared spectroscopy-IVUS as a reference with an area under the curve of 0.97 (95% CI, 0.93–1.00), and an optimal volume threshold of 2.30 mm^3^. In our study, the same AI-based analysis provided strong correlations for the quantification of vessel, lumen, and plaque volumes in segments of non-calcified plaque (Pearson’s correlations of 0.95, 0.97, and 0.83, respectively) and attenuated plaques (Pearson’s correlations of 0.90, 0.86, and 0.86, respectively).

Because it is not possible to visualize and characterize tissue behind an attenuated plaque on IVUS, we computed the entire plaque area and volumes on segments containing attenuated plaques, and not the volume of the attenuated component. This contrasts with previous IVUS analysis, which assumed that the tissue behind the superficial plaque attenuation was entirely attenuated, partly explaining the poor AI-CCTA and IVUS correlation for attenuated plaque volume (Pearson’s correlation, 0.28) [8].

Similarly, because IVUS does not allow tissue characterization behind the leading surface of calcium, we did not assume the entire plaque volume was calcified. Rather, we calculated a previously reported calcium index that indirectly represents the total amount of calcium per coronary segment [17], a metric that correlated with ex vivo histologic quantification of coronary calcium [22]. We found a strong correlation between AI-QCT and IVUS for the quantification of calcium length, angle, and calcium index.

Our study included continuous vascular segments unrestricted to specific plaques or plaque burden thresholds. Therefore, it was expected to find normal vessel segments interspersed between diseased segments; 30 such segments were found in this dataset. This exposed a methodological difference in how IVUS and CCTA quantify normal vessels. While IVUS detects the intima-media complex distinct from the lumen, CCTA’s limited spatial resolution superimposes the lumen and outer vessel wall contours (Supplementary Fig. 5). This resulted in IVUS reporting an apparent “plaque burden” of 26.9 ± 4.3% in normal segments, while AI-QCT measured only 4.8 ± 10.6%. This systematic difference must be interpreted considering the methodological differences between the two modalities, rather than measurement error by either technique.

### Stenosis quantification

Quantification of stenosis severity complements the prognostic value of atherosclerotic burden for guiding therapeutic decisions [23]. In clinical practice, overestimating stenosis severity is not uncommon, particularly among less experienced readers. This was confirmed in an analysis of 4347 patients in the PROMISE study, where core laboratory and site interpretations were discordant in 16%, mostly by sites reporting significant CAD, not confirmed by the core laboratory [24].

In the current study, AI-QCT underestimated the MLA by only 0.61 mm^2^ vs IVUS. Although modest, this MLA underestimation must be considered when decisions are based on dichotomous MLA thresholds. However, when the MLA values were compared with the lumen areas at the most normal reference sites—adjusting the absolute MLA to the actual vessel size—AI-QCT showed excellent agreement with IVUS for lumen area stenosis (mean difference, 1.26 ± 24.17%; 95% CI, −3.37% to 5.90%).

Remarkably, MLA sites were independently determined by each modality during automated data post-processing rather than actively co-registered. AI-QCT automatically identified MLA locations within 1.79 mm of IVUS determinations. This spatial agreement demonstrates AI-QCT’s capability for automatic lesion localization, supporting its use for objective stenosis assessment.

### Potential clinical implications of our findings

Validating AI-QCT across the entire spectrum of atheroma burden, including normal, mild, and advanced disease, provides clinicians with accurate data for assessment of cardiovascular risk, individualized preventive strategies, and guiding therapeutic strategies. This whole-vessel validation addresses a critical gap in current AI validation methodology, as clinical decisions increasingly require accurate plaque quantification across all disease severity levels rather than just focal high-risk lesions. The narrow 95% limits of agreement and consistency across disease severities support AI-QCT’s application for atheroma quantification.

Our findings complement recent evidence showing that comprehensive plaque characterization, including assessment of high-risk plaque features and quantitative plaque burden quantification, enhances cardiovascular risk prediction and improves discrimination of ischemia-causing lesions [25, 26], though clinical adoption has been limited by inconsistent reporting and reader variability in manual assessment [27].

The standardized, automated AI-based CCTA analysis has the potential to democratize comprehensive plaque assessment by eliminating the expertise-dependent variability that has limited its clinical adoption. By providing rapid, reproducible quantification with minimal inter-observer variation, AI-based CCTA analysis can bridge the gap between experienced subspecialty readers and general radiologists and cardiologists, making accurate atherosclerosis assessment accessible in routine practice. This standardization is particularly valuable for monitoring disease progression and therapeutic response, where consistent and reproducible quantification of the total atherosclerosis burden is key.

Automated CCTA workflows integrating a per-vessel quantification of atheroma burden, lumen stenosis, high-risk plaque features, and the presence and distribution of ischemia support disease phenotyping, therapeutic planning, and treatment monitoring.

### Limitations

Our study has several limitations. First, although we analyzed continuous coronary segments rather than selecting high-grade stenoses, the mean co-registered length (38.5 mm) was limited by the length of clinically acquired IVUS pullbacks rather than following a specific full-vessel imaging protocol. Nonetheless, these segments are longer than those in previous reports restricted to selected stenoses [8]. Second, our multicenter design introduced heterogeneity in CT scanners (four vendors), acquisition protocols (tube voltage: 100–120 kV), and contrast agents, which could have potentially affected plaque attenuation and lumen measurements. However, this variability reflects real-world practice. Furthermore, AI-QCT was trained on a diverse, vendor-agnostic dataset. Whether newer high-slice-count scanners beyond the 64-slice systems used in our analysis would further improve AI-QCT accuracy requires future investigation. Third, while different IVUS systems with 40-MHz and 60-MHz catheters were used per institutional availability, prior validation studies demonstrated these frequencies provide comparable quantitative measurements, with relative differences below 10% and within clinically acceptable limits [28, 29]. Furthermore, all IVUS images were analyzed using the same software and measurement protocol by a single senior operator with high reproducibility (Supplementary Table 2), ensuring standardized analysis independent of catheter frequency. Fourth, using IVUS as a reference precluded characterizing tissue behind superficial attenuation and calcium, preventing validation of attenuated and calcified volumes and their relative percentage of the total plaque volume, common CCTA metrics. Instead, we reported the total plaque volume in segments of attenuated plaques and calculated a previously validated calcium index. In normal coronary segments, IVUS quantifies the intima-media complex as “plaque,” while CCTA lacks the resolution to resolve the vessel wall thickness (Supplementary Fig. 5). Despite these methodological differences, AI-QCT demonstrated strong correlations with IVUS for the quantification of plaque volume and PAV; had the analysis been limited to selected regions with high plaque burden, one would expect even stronger correlations. Fifth, we did not systematically document the frequency and type of manual corrections during the AI-QCT quality review. As part of the FDA-approved clinical workflow, all AI-QCT outputs undergo mandatory review by a qualified radiology technologist who verifies centerline placement, vessel identification and labeling, and the automated lumen and outer wall contours. When necessary, manual adjustments are performed to correct centerline deviations, vessel mislabeling, or refine lumen and outer wall boundaries in segments affected by artifacts, heavy calcification, motion, or low contrast. This workflow has been extensively validated [13, 30–32], and it reflects the FDA-cleared clinical workflow used in routine patient care across hospitals offering this service, rather than a controlled research-only pipeline with predefined logging of user interventions. Sixth, our results are specific to the tested AI-QCT platform and cannot be extrapolated to other AI-based CCTA analysis systems. Finally, prognostic implications of our findings were beyond the scope of our study and warrant future investigation with adequate power for assessment of clinical outcomes.

## Conclusions

In the multicenter INVICTUS registry, AI-QCT showed high correlations and close agreement with a blinded IVUS core laboratory quantification of mean plaque burden, PAV, and length-normalized PAV along whole co-registered vascular segments, and at segments containing non-calcified and attenuated plaques. AI-QCT demonstrated high agreement with IVUS in localizing the MLA site and quantifying the lumen area stenosis without human interaction. These results support the incorporation of AI-QCT into clinical practice as a fast, reproducible, and accurate tool for quantification of total atherosclerotic burden and quantification of stenosis severity.

## Supplementary information

Supplementary information

## Abbreviations

AI: Artificial intelligence
AI-QCT: Artificial intelligence-based coronary computed tomography angiography quantification
CAD: Coronary artery disease
CCTA: Coronary computed tomography angiography
CI: Confidence interval
EEM: External elastic membrane
HU: Hounsfield units
INVICTUS: INVasive and non-Invasive imaging modalities to compare coronary Computed Tomography angiography, intravascular Ultrasound, and optical coherence tomography for the determination of severity, volume, and type of coronary atherosclerosis
IVUS: Intravascular ultrasound
MLA: Minimum lumen area
PAV: Percent atheroma volume
SD: Standard deviation
