# Supplementary material for: Artificial intelligence-based coronary computed tomography angiography quantification of atherosclerosis burden: comparison with intravascular ultrasound in the INVICTUS Registry
Source: Eur Radiol. 2026 Mar 5;36(7):5364–77. doi: 10.1007/s00330-026-12412-y (PMC13282242; doi:10.1007/s00330-026-12412-y)
Supplement: Supplementary file 1 — Supplementary information [file 330_2026_12412_MOESM1_ESM.pdf]

Artificial Intelligence-Based Coronary Computed Tomography Angiography Quantification of Atherosclerosis Burden: Comparison With Intravascular Ultrasound in the INVICTUS Registry

ELECTRONIC SUPPLEMENTARY MATERIAL

Eur Radiol (2026) Nakanishi R, Okubo R, Matsuo H, et al.

Table of Contents

SUPPLEMENTAL TABLE 1. STARD (STANDARDS FOR REPORTING OF DIAGNOSTIC ACCURACY) CHECKLIST. 3

SUPPLEMENTAL TABLE 2. INTRA-OBSERVER VARIABILITY DATA FROM A SUBSET OF 20 IVUS CASES FROM THE SINGLE SENIOR IVUS ANALYST. 7

SUPPLEMENTAL TABLE 3. DEFINITIONS OF PLAQUE CHARACTERIZATION BY IVUS AND CCTA AND FINAL PLAQUE PHENOTYPE 8

SUPPLEMENTAL TABLE 4. AI-QCT AND IVUS QUANTIFICATION OF PLAQUE VOLUME IN VESSEL SEGMENTS STRATIFIED ACCORDING TO CCTA-DERIVED DIAMETER STENOSIS 9

SUPPLEMENTAL TABLE 5. AI-QCT AND IVUS QUANTIFICATION OF PLAQUE VOLUME IN VESSEL SEGMENTS STRATIFIED ACCORDING TO CCTA-DERIVED PLAQUE STAGES. 10

SUPPLEMENTAL FIGURE 1. EXAMPLES AND DEFINITIONS OF PLAQUE CHARACTERIZATION BY IVUS AND CCTA 11

SUPPLEMENTAL FIGURE 2. LIMITATIONS OF DETERMINING THE AREA OF ATTENUATED PLAQUE COMPONENT ON IVUS IMAGES 12

SUPPLEMENTAL FIGURE 3. LIMITATIONS OF DETERMINING THE AREA OF CALCIFIED PLAQUES ON IVUS IMAGES 13

SUPPLEMENTAL FIGURE 4. CALCULATION OF THE CALCIUM INDEX BY IVUS 14

SUPPLEMENTAL FIGURE 5. METHODOLOGY DIFFERENCES BETWEEN IVUS AND CCTA IN THE ASSESSMENT OF NORMAL CORONARY ARTERIES 15

SUPPLEMENTAL FIGURE 6. SCATTERPLOTS AND BLAND-ALTMAN GRAPHS FOR THE QUANTIFICATION OF PLAQUE VOLUME IN SEGMENTS STRATIFIED BY CCTA-DERIVED DIAMETER STENOSIS 16

SUPPLEMENTAL FIGURE 7. SCATTERPLOTS AND BLAND-ALTMAN GRAPHS FOR THE QUANTIFICATION OF PLAQUE VOLUME IN SEGMENTS STRATIFIED BY CCTA-DERIVED PLAQUE STAGES 17

Supplemental Table 1. STARD (Standards for Reporting of Diagnostic Accuracy) Checklist.

This is a quantitative method comparison study evaluating measurement agreement between AI-based coronary CT angiography quantification (AI-QCT) and intravascular ultrasound (IVUS) using correlation analysis and Bland-Altman plots. While not primarily a diagnostic accuracy study in the traditional sense (sensitivity/specificity for disease detection), we have completed applicable STARD items. Items specific to binary diagnostic classification are marked N/A with explanations.

Section	Item	Reporting Item	Page/Location	Notes
**TITLE/ABSTRACT**				
	1	Identification as a study of diagnostic accuracy using at least one measure of accuracy (such as sensitivity, specificity, predictive values, or AUC)	Title Page, Abstract	Correlation and agreement (Bland-Altman) measures provided rather than traditional diagnostic accuracy metrics, as they are appropriate for continuous variable comparison.
	2	Structured summary of study design, methods, results, and conclusions	Page 2, Abstract	Full structured abstract provided with Objectives, Material & Methods, Results and Conclusions.
**INTRODUCTION**				
	3	Scientific and clinical background, including the intended use and clinical role of the index test	Pages 3-4, Introduction, paragraphs 1-3	Clinical role of AI-QCT for atherosclerosis quantification and cardiovascular risk assessment described
	4	Study objectives and hypotheses	Page4, Introduction	
**METHODS – Study Design**				
	5	Whether data collection was planned before the index test and reference standard were performed (prospective study) or after (retrospective study)	Page 5, Methods, Study Design section	INVICTUS has a mixed design with retrospective and prospective patients included from January 2011 to December 2021. Cases included in the current analysis were randomly selected from the INVICTUS registry database
**METHODS – Participants**				
	6	Eligibility criteria	Pages 5-6, Methods, Study Design section	
	7	On what basis potentially eligible participants were identified	Page 5, Methods	
	8	Where and when potentially eligible	Pages 1-2 (affiliations),	17 centers in Japan from January 2011 to December 2021. Cases included in this

		participants were identified	Page 5, Methods	analysis were randomly selected from the INVICTUS registry database
	9	Whether participants formed a consecutive, random or convenience series	Page 5, Methods; Figure 2	Consecutive/convenience series based on availability of both CCTA and IVUS Imaging meeting quality criteria.
**METHODS – Test Methods**				
	10	Index and reference tests in sufficient detail to allow replication	Pages 6-7, Methods	Detailed protocols for both CCTA (scanner types, protocols, contrast, reconstruction) and IVUS (catheter and system types, pullback speeds, analysis software) provided. AI-QCT workflow described with reference to FDA-approved process.
	11	Rationale for choosing the reference standard	Page 4, Introduction; Page 5, Methods	IVUS chosen because it provides high-resolution intravascular visualization of coronary artery lumen and external elastic membrane, allowing for quantification of coronary plaque.
	12	Definition of and rationale for test positivity cut-offs or result categories of the index and reference tests, distinguishing pre-specified from exploratory	Pages 7-8, Methods, Analysis section; Supplemental Table 2	N/A for binary classification. However, plaque characterization categories were defined: low-attenuation (<30 HU), non-calcified (30-350 HU), and calcified (>350 HU) for CCTA; corresponding categories were provided for IVUS. Definition of minimum plaque área threshold for CCTA identification of plaque: $\geq 1mm^2$
	13	Whether clinical information and reference standard results were available to the performers / readers of the index test; Whether clinical information and index test results were available to the assessors of the reference standard	Page 7, Methods, Analysis section	Blinding explicitly stated: "The CCTA and IVUS core laboratories conducted the Imaging analysis independently and were blinded to each other's findings".
	14	Methods for estimating or comparing measures of diagnostic accuracy	Page 8, Methods, Statistical Analysis	Pearson's correlation and linear regression for association; Bland-Altman analysis for agreement.

	15	How indeterminate index test or reference standard results were handled	Pages 6-7, Methods; Figure 2	Quality control process described: "Only CCTA images graded as excellent or good quality were included in the analysis". Poor quality images were excluded. No indeterminate results in final analysis cohort.
	16	How missing data on the index test and reference standard were handled	Figure 2, Page 6-7, Methods	Vessels with missing or inadequate data or the lack of reliable anatomical landmarks were excluded during the co-registration process.
	17	Any analyses of variability in diagnostic accuracy, distinguishing pre-specified from exploratory	Page 8, Methods; Supplemental Table 2	Intra-observer reproducibility for IVUS reported (Supplemental Table 1: ICC 0.98-0.99 for key IVUS parameters). Subsegmental analyses (non-calcified, low attenuation, normal segments) were pre-specified based on clinical relevance.
	18	Intended sample size and how it was determined	Page 8, Methods, Statistical Analysis	
**RESULTS – Participants**				
	19	Flow of participants, using a diagram	Figure 2	
	20	Baseline demographic and clinical characteristics of participants	Page 9, Table 1	
	21	Distribution of severity of disease in those with the target condition, and distribution of alternative diagnoses in those without the target condition	Pages 9-11; Tables 2-4; Supplemental Tables 3-4	Disease severity distribution: mean plaque burden 51.3% (IVUS), range from normal segments to severe stenoses (mean MLA 2.3±1.9 mm²). Subsegmental analyss included 168 non-calcified segments, 37 low-attenuation segments, and 30 normal segments. Distribution by stenosis severity in Supplemental Table 3.
	22	Time interval and any clinical interventions between index test and reference standard	Page 9, Results, first paragraph	
**RESULTS – Test Results**				
	23	Cross tabulation of the index test results (or their distribution)	Table 3, Page 11	For plaque type classification at MLA site: agreement in 99.1% of cases (Kappa 0.98, 95% CI: 0.96-1.00). Cross-

		by the results of the reference standard		tabulation shows: 66/108 non-calcified (both modalities), 41/108 calcified (both modalities), 1/108 normal (both modalities). Within non-calcified: 20 vs. 21 fibrofatty/lipid by AI-QCT vs. IVUS
	24	Estimates of diagnostic accuracy and their precision (such as 95% confidence intervals)	Tables 2-4, Figures 3-5	Study reports correlation coefficients and agreement (Bland-Altman) measures rather than traditional diagnostic accuracy.
	25	Any adverse events from performing the index test or the reference standard	N/A	Not applicable. Study analyzed existing images clinically acquired. No adverse events were study-specific outcomes.
**DISCUSSION**				
	26	Study limitations, including sources of potential bias, statistical uncertainty, and generalisability	Pages 14-15, Limitations section	
	27	Implications for practice, including the intended use and clinical role of the index test	Pages 13-14, Discussion section, 'Potential Clinical Implications' sub-section	
**OTHER INFORMATION**				
	28	Registration number and name of registry	Page 1, Title page; Page 5, Methods	
	29	Where the full study protocol can be accessed	Page 4, Introduction; Reference 10	
	30	Sources of funding and other support; role of funders	Page 1, Title page, Funding section; Page 8, Methods, 'Role of the Sponsor' sub-section	

Supplemental Table 2. Intra-Observer Variability Data from a Subset of 20 IVUS Cases from the Single Senior IVUS Analyst.

**IVUS Variable**	**ICC**
Vessel (EEM) Area	0.9999
Mean Vessel (EEM) Diameter	0.9999
Lumen Area	0.9996
Minimum Lumen Area	0.9947
Mean Lumen Diameter	0.9996
Lumen Eccentricity Index	0.9949
Plaque Area	0.9996
Plaque Burden	0.9996
Plaque Max Thickness	0.9944
Plaque Min Thickness: Mean	0.9987

EEM: external elastic membrane

ICC values indicate the proportion of total measurement variability attributable to differences between subjects rather than observer inconsistency. It ranges from low ICC (close to 0; most variability comes from measurement error/observer inconsistency; unreliable measurement) to high (close to 1.0; most variability comes from real differences between subjects; measurement is reliable). Common interpretation is: ICC <0.40 (poor), 0.40–0.59 (fair), 0.60–0.74 (good), and 0.75–1.0 (excellent) agreement. The very high ICCs reported here demonstrate excellent intra-observer reliability of IVUS quantifications relevant to this study. Other derived metrics, such as atheroma volume, are calculated from these primary measurements.

Supplemental Table 3. Definitions of Plaque Characterization by IVUS and CCTA and Final Plaque Phenotype

**Plaque Characterization (IVUS)**	**Fibrotic**	**Soft**	**Attenuated**	**Calcified**
	echogenicity similar to the adventitia, and intermediate between soft and calcified plaque	Echogenicity lower than the adventitia	Low echogenicity with deep ultrasound attenuation without superficial calcium or dense fibrosis	Higher echogenicity than the adventitia; appear as bright echoes that cast shadow onto the posterior vascular wall.
**Plaque Characterization (CCTA)**	**Non-calcified**	**Low Attenuation**		**Calcified**
	Within-plaque Hounsfield Units range from 30 to 350	Within-plaque Hounsfield Units below 30		Within-plaque Hounsfield Units greater than 350
**Plaque Phenotype**	**Non-Calcified**			**Calcified**
	**Fibrotic**	**Lipid/Fibrofatty**		

IVUS: intravascular ultrasound; CCTA: coronary computed tomography angiography

Supplemental Table 4. AI-QCT and IVUS Quantification of Plaque Volume in Vessel Segments Stratified According to CCTA-derived Diameter Stenosis

	CCTA		IVUS		Paired Difference	
	Mean ± SD	95% CI	Mean ± SD	95% CI	Mean ± SD	95% CI
**All Sub-segments**						
DS 40-90% (n=172)	25.6 ± 38.2	19.9 to 31.4	32.4 ± 37.3	26.8 to 38.1	-6.8 ± 22.2	-10.2 to -3.5
DS <50% (n=74)	20.6 ± 25.7	14.7 to 26.6	30.8 ± 30.6	23.7 to 37.9	-10.1 ± 18.1	-14.3 to -5.9
DS 50-69% (n=84)	22.2 ± 28.0	16.2 to 28.3	31.5 ± 35.3	23.8 to 39.2	-9.3 ± 14.2	-12.4 to -6.2
DS ≥70% (n=73)	32.6 ± 49.1	21.1 to 44.1	36.7 ± 42.6	26.8 to 46.7	-4.1 ± 29.9	-11.1 to 2.8
**Non-Calcified Segments**						
DS 40-90% (n=123)	28.0 ± 41.7	26.6 to 38.9	35.1 ± 40.9	31.0 to 45.1	-7.0 ± 23.6	-7.4 to -3.2
DS <50% (n=55)	26.4 ± 27.3	19.0 to 33.7	34.6 ± 32.5	25.8 to 43.4	-8.3 ± 16.0	-12.6 to -3.9
DS 50-69% (n=64)	26.0 ± 30.3	18.5 to 33.6	37.0 ± 37.8	27.6 to 46.5	-11.0 ± 14.1	-14.5 to -7.5
DS ≥70% (n=49)	33.4 ± 55.6	17.4 to 49.4	38.4 ± 48.6	24.5 to 52.4	-5.0 ± 35.5	-15.2 to 5.2
**Segments with Attenuated Plaques**						
DS 40-90% (n=32)	29.9 ± 28.4	19.7 to 40.1	32.0 ± 25.0	23.0 to 41.0	-2.1 ± 14.7	-7.4 to 3.2
DS <50% (n=6)	12.9 ± 5.3	7.3 to 18.4	14.7 ± 9.1	5.1 to 24.3	-1.83 ± 6.94	-9.11 to 5.45
DS 50-69% (n=11)	18.2 ± 12.1	10.1 to 26.3	21.4 ± 18.4	9.1 to 33.8	-3.2 ± 18.1	-15.4 to 9.0
DS ≥70% (n=20)	37.2 ± 33.0	21.7 to 52.6	38.1 ± 27.1	25.4 to 50.8	-0.92 ± 13.09	-7.05 to 5.21

CCTA: coronary computed tomography angiography; IVUS: intravascular ultrasound; SD: standard deviation; CI: confidence interval; DS: diameter stenosis

Supplemental Table 5. AI-QCT and IVUS Quantification of Plaque Volume in Vessel Segments Stratified According to CCTA-derived Plaque Stages.

	CCTA		IVUS		Paired Difference	
	Mean ± SD	95% CI	Mean ± SD	95% CI	Mean ± SD	95% CI
**All Sub-segments**						
Plaque Stage 1 (n=28)	22.3 ± 17.2	15.6 to 28.9	26.7 ± 21.1	18.5 to 34.8	-4.4 ± 6.7	-7.0 to -1.8
Plaque Stage 2 (n=119)	28.5 ± 43.1	20.6 to 36.3	36.0 ± 39.5	28.8 to 43.2	-7.5 ± 24.7	-12.0 to -3.1)
Plaque Stage 3 (n=84)	21.0 ± 27.5	15.0 to 27.0	30.6 ± 35.6	22.9 to 38.4	-9.6 ± 20.0	-14.0 to -5.3
**Non-Calcified Segments**						
Plaque Stage 1 (n=21)	27.1 ± 17.1	19.3 to 34.9	31.9 ± 21.8	22.0 to 41.8	-4.8 ± 7.5	-8.2 to -1.4
Plaque Stage 2 (n=92)	31.7 ± 47.0	22.0 to 41.5	40.2 ± 42.8	31.3 to 49.1	-8.5 ± 27.1	-14.1 to -2.9
Plaque Stage 3 (n=55)	23.0 ± 26.8	15.8 to 30.3	32.5 ± 39.0	22.0 to 43.1	-9.5 ± 19.2	-14.7 to -4.3
**Segments with Attenuated Plaques**						
Plaque Stage 1 (n=5)	10.9 ± 3.7	6.2 to 15.5	13.4 ± 5.8	6.2 to 20.6	-2.5 ± 2.7	-5.9 to 0.9
Plaque Stage 2 (n=14)	33.5 ± 22.3	20.5 to 46.4	31.4 ± 21.1	19.2 to 43.5	2.1 ± 11.4	-4.5 to 8.7
Plaque Stage 3 (n=18)	27.7 ± 32.5	11.5 to 43.8	32.2 ± 28.6	18.0 to 46.4	-4.5 ± 16.7	-12.8 to 3.8

CCTA: coronary computed tomography angiography; IVUS: intravascular ultrasound; SD: standard deviation; CI: confidence interval.

CCTA-derived plaque stages were defined as: Stage 1 (mild plaque): percent atheroma volume (PAV) >0-5%; Stage 2 (moderate plaque): PAV >5-15%; Stage 3 (severe plaque): PAV >15% (Min JK et al. J Cardiovasc Comput Tomogr. 2022 Sep-Oct;16(5):415-422. doi: 10.1016/j.jcct.2022.03.001).

Supplemental Figure 1. Examples and Definitions of Plaque Characterization by IVUS and CCTA

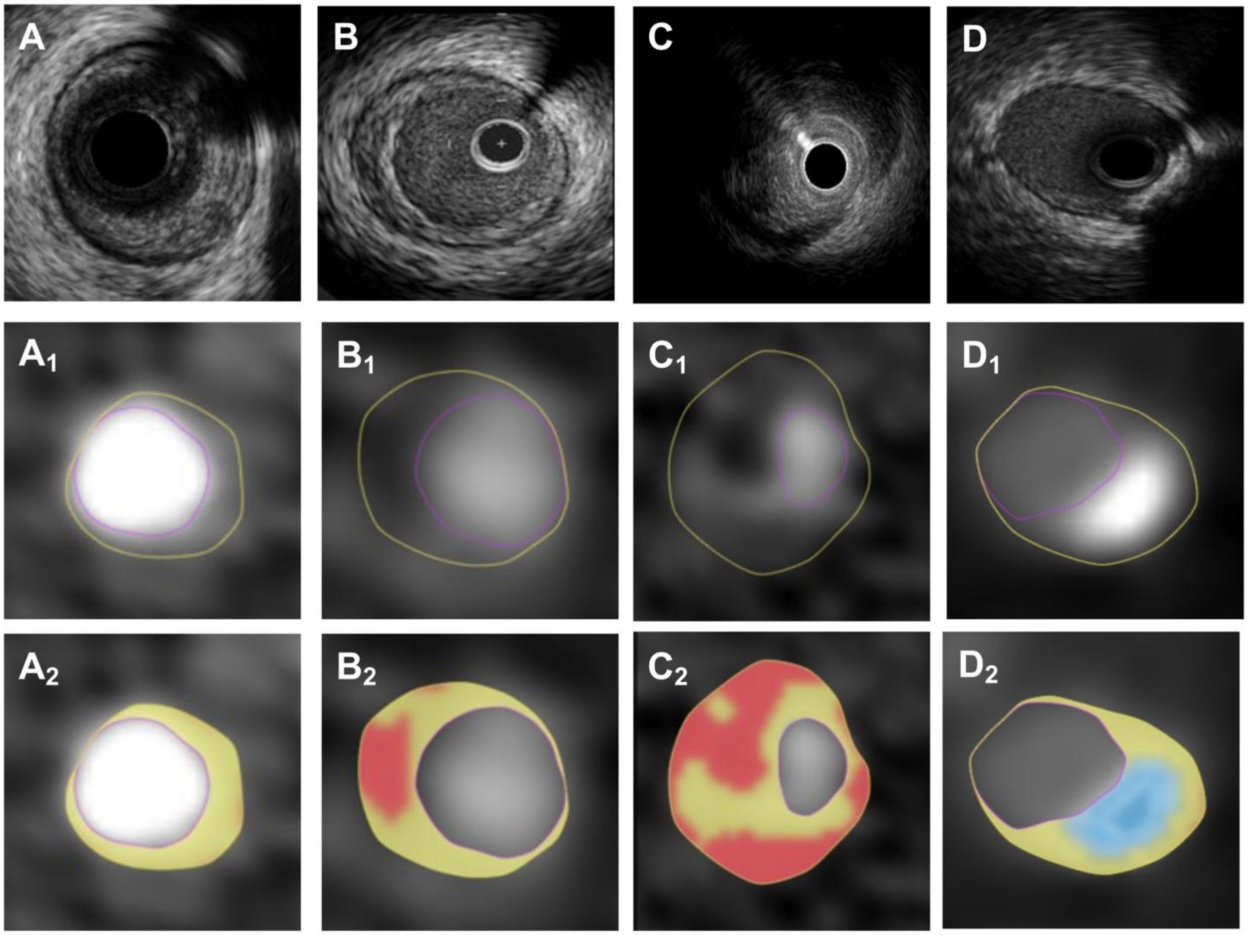

By IVUS, fibrotic plaques (A) are characterized by having an intermediate echogenicity between the soft and calcified plaque, similar to the adventitia. Soft or echolucent plaques (B) have an echogenicity lower than the adventitia. Highly attenuated plaques (C) are hypoechoic with deep ultrasound attenuation without superficial calcification or dense fibrosis. Calcified plaques (D) are highly echogenic and appear as a bright echo that casts a shadow onto posterior vascular structures. On CCTA, plaques are classified as non-calcified (A_1) when the within plaque Hounsfield units (HU) range from 30 to 350 HU and are color-coded yellow by the AI-QCT software (A_2). Low-attenuation tissue (B_1 and C_1) presents a HU below 30 and is color-coded red by the AI-QCT software (B_2 and C_2). Calcified plaques (D_1) present a HU greater than 350 and are color-coded blue by the AI-QCT software. Plaques represented by cross-sections A, B, and C were categorized as non-calcified. Of these, those displayed on cross-sections B and C were further classified as fibro-fatty/lipid.

Supplemental Figure 2. Limitations of Determining the Area of Attenuated Plaque Component on IVUS Images

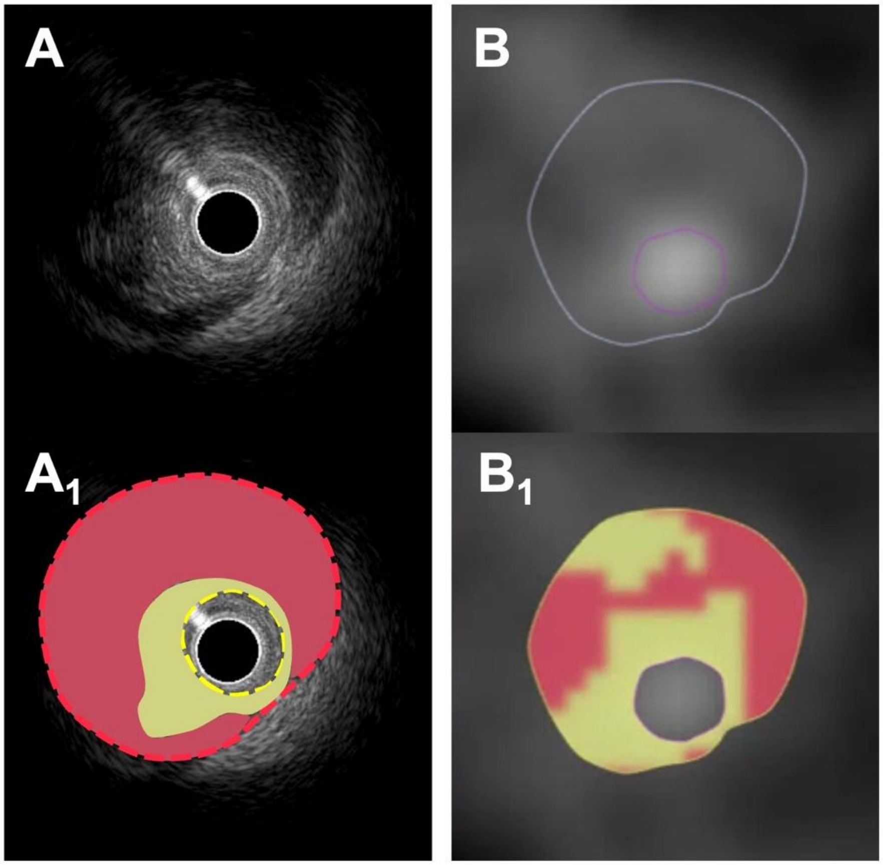

Panel A presents an IVUS cross-section of an attenuated plaque. Panel A_1 represents a simulation of plaque component quantification. The yellow shading delimits the visible superficial fibrotic part of the plaque, while the red shading assumes the entire plaque area behind the superficial attenuation is indeed attenuated. Panel B presents the corresponding CCTA cross-section of the same plaque. The low-attenuation component, characterized by a Hounsfield unit (HU) threshold <30 is color-coded red, while non-calcified tissue (30-350 HU) is color-coded yellow. By assuming that the entire plaque area behind the superficial attenuation is indeed attenuated on the IVUS images, one significantly overestimates the area and volume of the attenuated components as compared with CCTA. Thus, we refrained from such an analysis and reported the entire plaque volume at segments with attenuated plaques.

Supplemental Figure 3. Limitations of Determining the Area of Calcified Plaques on IVUS Images

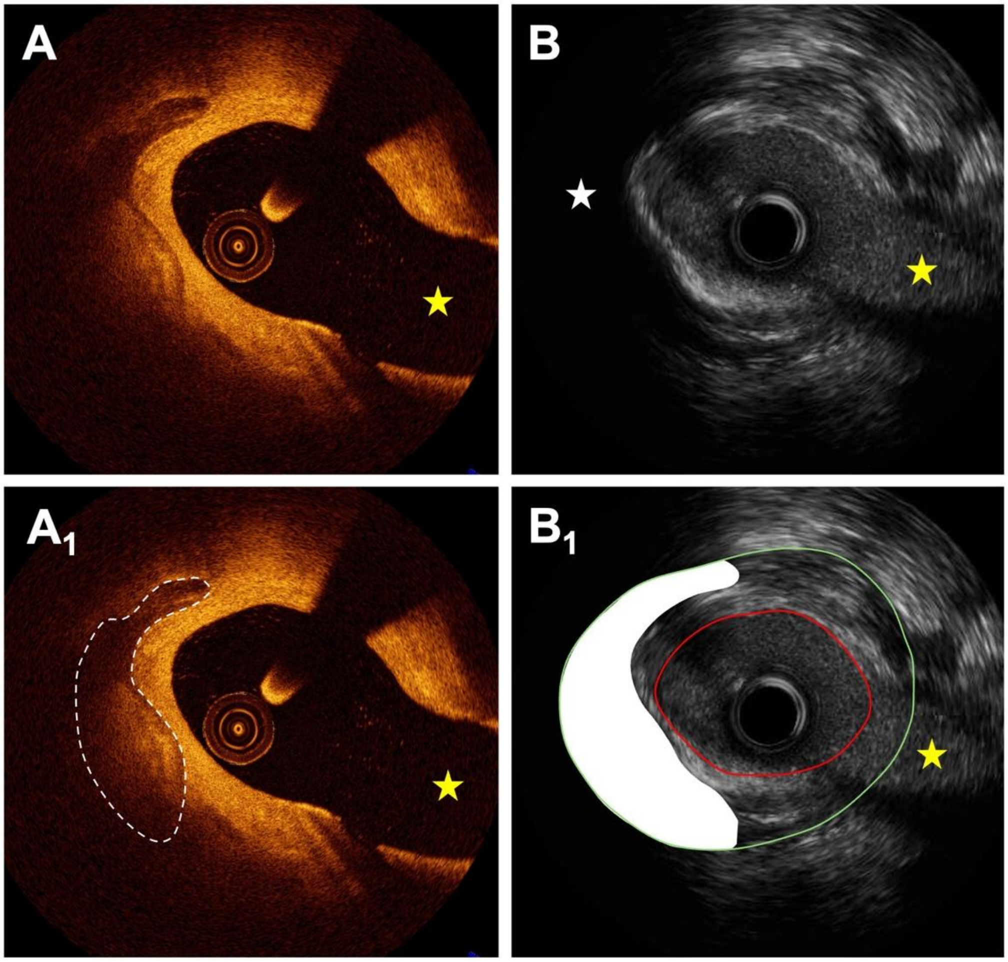

Panels A and B present an optical coherence tomography (OCT) and IVUS co-registered cross-sections acquired outside the INVICTUS registry, showing a calcified plaque opposite to a side branch (yellow stars). The infrared light emitted by the OCT catheter penetrates calcium and allows the determination of its area (dashed white contour in panel A_1). On IVUS, calcium appears as a bright echo that casts a shadow onto posterior vascular structures (white star in panel B). Assuming that the entire shadowed area is calcified (white shadowed area in panel B_1) would result in a significant overestimation of calcium dimensions by IVUS.

Supplemental Figure 4. Calculation of the Calcium Index by IVUS

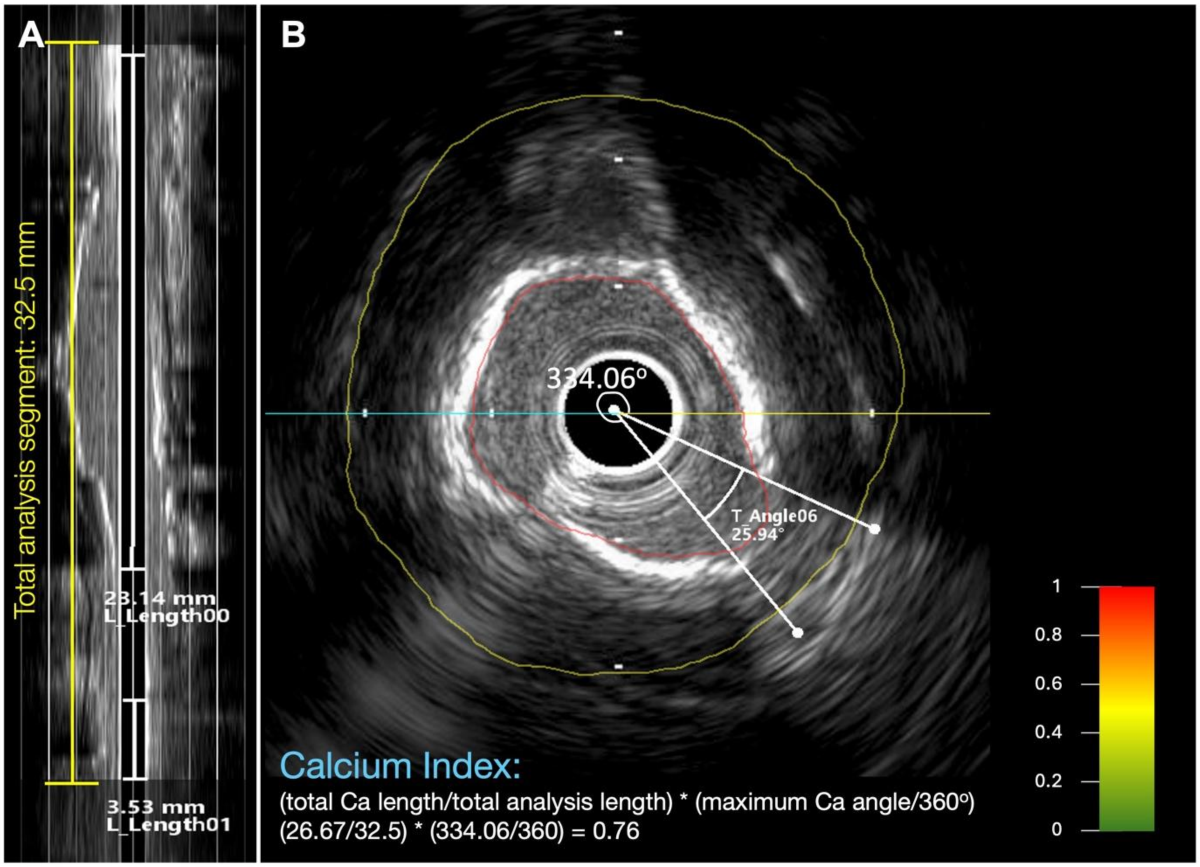

Due to the limitations presented in Supplemental Figure 3, we determined the calcium index to estimate the calcium burden on IVUS pullbacks. The calcium index was calculated as the total calcium length (the sum of consecutive calcium lengths solid white lines in panel A) divided by the total length of the analyzed segment (solid yellow line in panel A) multiplied by the maximum calcium arc (panel D) divided by 360º. The calcium index ranges from 0 (no calcium) to 1 (maximum calcium burden) (scale in the bottom right of panel B).

Supplemental Figure 5. Methodology Differences Between IVUS and CCTA in the Assessment of Normal Coronary Arteries

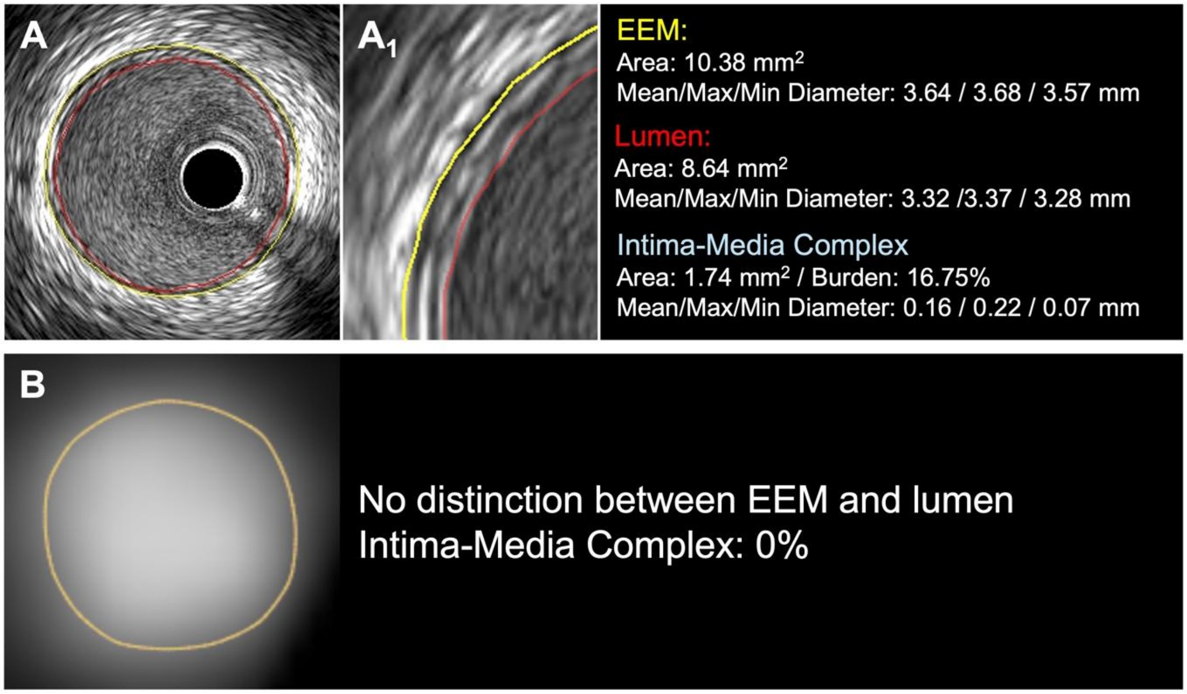

In normal coronary arteries, IVUS detects the intima-media complex that separates the lumen (red contour in panel A) from the EEM (yellow contour in panel A), misreporting the EEM minus lumen difference (see magnified image in panel A_1) as plaque burden. In the presented IVUS cross-section, the software miscalculates a plaque burden of 16.75%. The mean, maximum, and minimum thicknesses of the intima-media complex measured 0.16, 0.22, and 0.07 mm, respectively – figures well below the axial resolution of CCTA (on average 0.3 mm). As a result, CCTA does not resolve the normal coronary wall thickness, overlapping the EEM and lumen contours. This correctly results in no plaque computation and, thus, no plaque burden reporting in normal coronary segments.

Supplemental Figure 6. Scatterplots and Bland-Altman Graphs for the Quantification of Plaque Volume in Segments Stratified by CCTA-Derived Diameter Stenosis

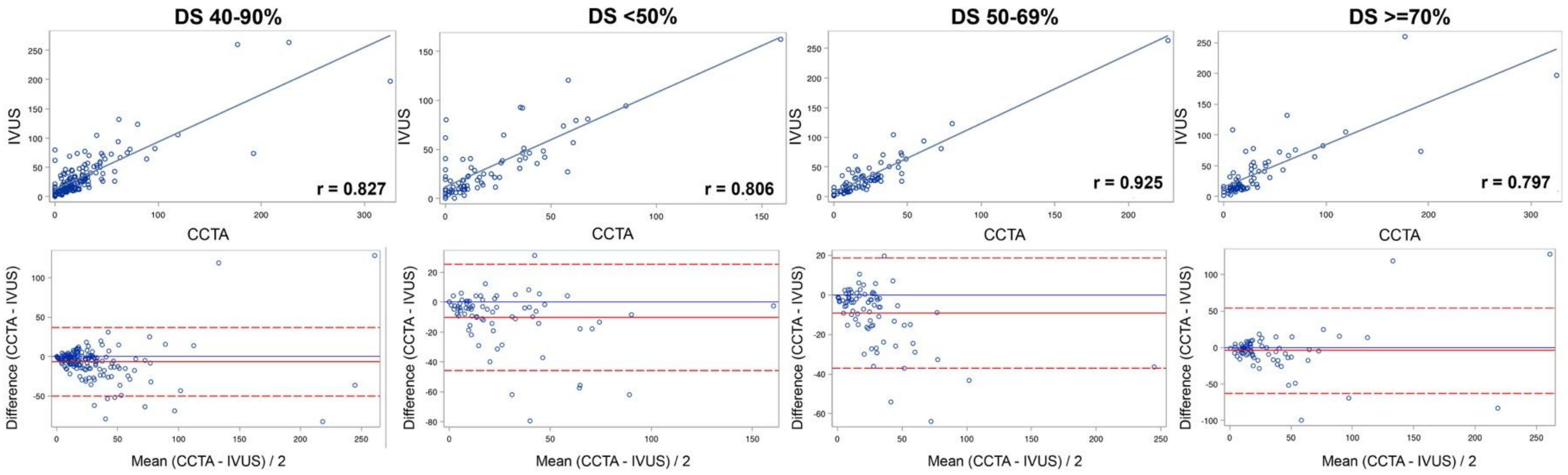

DS: diameter stenosis

Supplemental Figure 7. Scatterplots and Bland-Altman Graphs for the Quantification of Plaque Volume in Segments Stratified by CCTA-Derived Plaque Stages

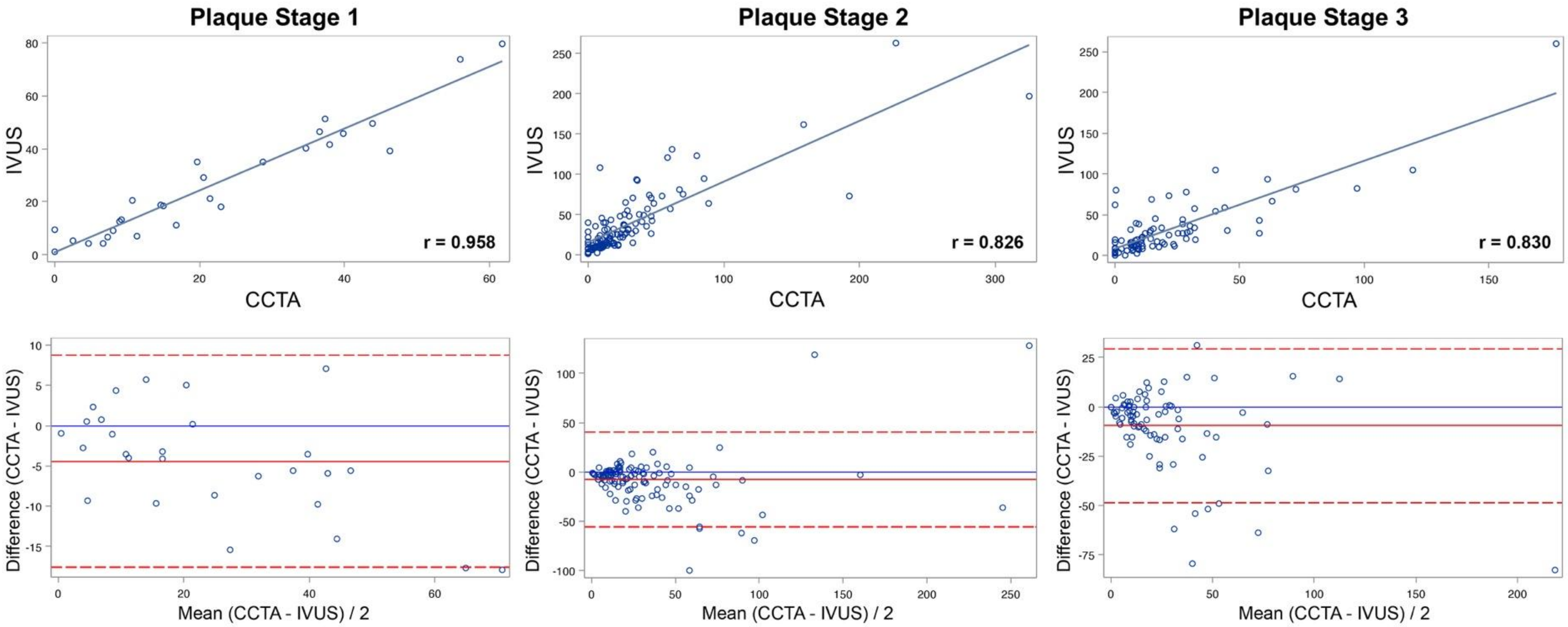

CCTA-derived plaque stages were defined as: Stage 1 (mild plaque): percent atheroma volume (PAV) >0-5%; Stage 2 (moderate plaque): PAV >5-15%; Stage 3 (severe plaque): PAV >15% (Min JK et al. J Cardiovasc Comput Tomogr. 2022 Sep-Oct;16(5):415-422. doi: 10.1016/j.jcct.2022.03.001).
